# Editorial: The Elderly Athlete

**DOI:** 10.3389/fphys.2021.686858

**Published:** 2021-05-07

**Authors:** Beat Knechtle, Romuald Lepers, Pantelis Theodoros Nikolaidis, Caio Victor Sousa

**Affiliations:** ^1^Medbase St. Gallen Am Vadianplatz, St. Gallen, Switzerland; ^2^Institute of Primary Care, University of Zurich, Zurich, Switzerland; ^3^CAPS UMR1093 INSERM, Faculty of Sport Sciences, University de Bourgogne-Franche Comté, Dijon, France; ^4^School of Health and Caring Sciences, University of West Attica, Athens, Greece; ^5^Bouve College of Health Sciences, Northeastern University, Boston, MA, United States

**Keywords:** swimming, running, cycling, endurance, age group

Age-related declines in physical activity levels and exercise training can accelerate the reductions in physical function and performance in older adults. Because elderly athletes (masters athletes) maintain high levels of physical activity, they may serve as an example of an optimal healthy human aging. Indeed, the master athlete has been proposed as an ideal model to determine successful aging due to his or her chronic participation in high-intensity exercise (Lazarus and Harridge, 2017; Tanaka et al., 2019).

The last decades had a remarkable increase in endurance and ultra-endurance events, reflecting a significant participation increase of master athletes. Master athletes compete not only for the glory of participation but also for the achievement of high performance.

In recent years, several astonishing reports show the outstanding achievements of master athletes (Lepers and Stapley, 2016). For example, master athletes compete in distance running (i.e., 100, 200, 400, 800, 1,500, 5,000, 10,000 m, and marathon) until the age group 95–99 years (Schneider et al., 2019). The number of master marathoners older than 75 years has increased in the last 25 years (Ahmadyar et al., 2016), and master marathoners can finish a marathon at the age of 90 years and older (Knechtle et al., 2014a; Mueller et al., 2014). In some instances, male master marathoners older than 90 years can complete a 6-h-run (Knechtle and Nikolaidis, 2018) or even a 12-h-run (Knechtle et al., 2018). Recently, it has been reported that master runners older than 80- or 90 years finished a 100-km ultra-marathon (Stohr et al., 2021).

It is even possible to achieve a World Record after the 100th year of age. A study by Lepers et al. (2016) investigated all best performances achieved by centenarians for swimming, cycling, and running. They found 60 performances belonging to 19 individuals, with 10 in athletics, 8 in swimming, and one in cycling.

Apart from running, swimming seems to be an ideal sports discipline for centenarians. An analysis of participation and performance of male and female age group backstroke swimmers competing in 50, 100, and 200 m pool swimming at the FINA World Masters Championships held between 1986 and 2014 showed that swimmers in age group 100–104 compete in this discipline (Unterweger et al., 2016). The Canadian Jaring Timmerman, the world's oldest masters swimmer, set four world records in the age group 100–104 years and stands solely responsible for creating the age group 105–109 years in masters swimming (Swam).

In this context, a total of seven articles were published- six studies investigated elderly athletes—in the Research Topic “The Elderly Athlete” (www.frontiersin.org/research-topics/14067/the-elderly-athlete#overview). These studies investigated different aspects such as muscle strength (Taveira et al.), cardiac characteristics (Wooten et al.; Hoffmann et al.), physical fitness, and lifestyle (Wooten et al.).

A study comparing trunk muscle strength and postural control of older adults (age ≥ 65 years old) found thatolder adult runners presented a higher isokinetic torque of extensor trunk muscles postural control than non-runners (Taveira et al.). Two studies explored cardiac aspects of master athletes. A first study investigated sex differences in cardiac structure, function, and left ventricular systolic global longitudinal strain among female and male masters athletes of 55–60 years old, showing cardiac sex differences regarding selected cardiac dimensions (Wooten et al.). A second study compared left ventricular dimensions and function in elite master athletes aged 60 years involved in throwing events requiring strength to those interested in endurance events and sprinting. Left ventricular diastolic function was not different in throwers but superior in endurance athletes and sprinters than age-matched historical controls (Hoffmann et al.). Physical fitness seems to affect also later in life. A study investigating 240 master athletes participating in the World Masters Athletics Championships showed that the lifestyles of master athletes contributed to improved general life satisfaction (Wooten et al.). Another study investigated the age-related decline in running performance of sub-3-h marathoners for five consecutive calendar decades longitudinally. The authors found that it is possible with consistent training and racing regimensto limit the age-related decline in marathon performance to < 7% per decade at least until 60 years of age (Lepers et al.).

Although athletes of the age of 60–65 years belong by definition to the category of master athletes, athletes at the age of 50 years are at their peak for very long (48-h run) ultra-marathons (Knechtle et al., 2014b). Runners older than 70 years finishedthe “Leadville 100-Mile Endurance Race” in Colorado, USA (Charles Williams at the age of 70 years), the “Badwater 135-Mile Ultramarathon Race” which is considered as the “Toughest Footrace In The World” in California, USA (Jack Deness, 75 years), the “UTMB” (Ultra-Trail du Mont-Blanc) in Chamonix, France (Christoph Geiger, 73 years) or the Western States 100-Mile Endurance Run in California, USA (Nick Bassett, 73 years) (Runner, 2019).

Finally, we thank all authors, reviewers, and editors for their contribution to the present Research Topic. We hope that this Research Topic will stimulate further research in this area. Future scientific studies need to investigate the motivation, preparation, and physiology of master athletes 70 years and older up to the centenarians.

To date scientific evidence in the field comes mainly from studies conducted in men only. Research is nevertheless warranted to determine potential sex differences in the effects of lifelong exercise on the age-related decline in performance. The aspect of sex differences in the elderly athlete is a challenging topic especially considering the increase of sports participation in women, e.g., the men-to-women ratio of finishers in the New York City Marathon decreased from 10.16 in the 1970s to 1.52 in the 2010s (Vitti et al., 2020).

## Author Contributions

All authors listed have made a substantial, direct and intellectual contribution to the work, and approved it for publication.

## Conflict of Interest

The authors declare that the research was conducted in the absence of any commercial or financial relationships that could be construed as a potential conflict of interest.
